# A cluster randomized trial for the implementation of an antibiotic checklist based on validated quality indicators: the AB-checklist

**DOI:** 10.1186/s12879-015-0867-2

**Published:** 2015-03-19

**Authors:** Frederike V van Daalen, Jan M Prins, Brent C Opmeer, Marja A Boermeester, Caroline E Visser, Reinier M van Hest, Marlies E J L Hulscher, Suzanne E Geerlings

**Affiliations:** Department of Internal Medicine, Division of Infectious Diseases, Academic Medical Centre, Meibergdreef 9, 1105 AZ Amsterdam, The Netherlands; Clinical Research Unit, Academic Medical Centre, Meibergdreef 9, 1105 AZ Amsterdam, Netherlands; Department of General Surgery, Academic Medical Centre, Meibergdreef 9, 1105 AZ Amsterdam, Netherlands; Department of Medical Microbiology, Academic Medical Centre, Meibergdreef 9, 1105 AZ Amsterdam, Netherlands; Department of Hospital Pharmacy, Academic Medical Centre, Meibergdreef 9, 1105 AZ Amsterdam, Netherlands; Department of IQ healthcare, Radboud University Medical Centre, Geert Grooteplein-Zuid 10, 6525 GA Nijmegen, Netherlands

**Keywords:** Checklist, Antibiotics, Implementation, Quality indicators, Stepped-wedge design

## Abstract

**Background:**

Recently we developed and validated generic quality indicators that define ‘appropriate antibiotic use’ in hospitalized adults treated for a (suspected) bacterial infection. Previous studies have shown that with appropriate antibiotic use a reduction of 13% of length of hospital stay can be achieved. Our main objective in this project is to provide hospitals with an antibiotic checklist based on these quality indicators, and to evaluate the introduction of this checklist in terms of (cost-) effectiveness.

**Methods/Design:**

The checklist applies to hospitalized adults with a suspected bacterial infection for whom antibiotic therapy is initiated, at first via the intravenous route. A stepped wedge study design will be used, comparing outcomes before and after introduction of the checklist in nine hospitals in the Netherlands. At least 810 patients will be included in both the control and the intervention group. The primary endpoint is length of hospital stay. Secondary endpoints are appropriate antibiotic use measured by the quality indicators, admission to and duration of intensive care unit stay, readmission within 30 days, mortality, total antibiotic use, and costs associated with implementation and hospital stay. Differences in numerical endpoints between the two periods will be evaluated with mixed linear models; for dichotomous outcomes generalized estimating equation models will be used. A process evaluation will be performed to evaluate the professionals’ compliance with use of the checklist. The key question for the economic evaluation is whether the benefits of the checklist, which include reduced antibiotic use, reduced length of stay and associated costs, justify the costs associated with implementation activities as well as daily use of the checklist.

**Discussion:**

If (cost-) effective, the AB-checklist will provide physicians with a tool to support appropriate antibiotic use in adult hospitalized patients who start with intravenous antibiotics.

**Trial registration:**

Dutch trial registry: NTR4872

## Background

### The need to improve antibiotic use

The increasing antimicrobial resistance rate is one of the most important health care problems at this moment. The total consumption of antibiotics is the main driving force [1,2]. The World Health Organization signalled the emergence of antimicrobial resistance (AMR), along with the steady decline in the discovery of new antimicrobials, as a major health threat for the coming decade. To help control AMR, a better use of the current agents is necessary [3]. Recent studies have shown considerable room for improvement in the two most common bacterial infections: respiratory and urinary tract infections [4,5]. An important question is how to achieve such an improvement.

Previous studies have shown that appropriate antibiotic use is not only of great importance to curb antimicrobial resistance, but also has a short-term consequence. The evaluation of antibiotic treatment for urinary tract infections in hospitals showed an inverse relationship between the proportion of appropriate antibiotic use in a patient and length of stay (LOS). Prescribing therapy in accordance with local hospital guidelines was associated with a shorter LOS (7.3 days vs. 8.7 days; P = 0.024) [6]. Recently a similar inverse relationship has been shown between appropriate antibiotic use in treatment of all bacterial infections in the hospital and LOS, with a reduction of 13% in LOS with appropriate use. [*Abstract poster:* Quality Indicators for Monitoring Appropriate Antibiotic Use in Hospitals: an Important Tool for Antibiotic Stewardship. K-325. *54th Interscience Conference on Antimicrobial Agents and Chemotherapy, September 5 -9, 2014, Washington DC*].

### Quality indicators

Guidelines defining appropriate antimicrobial treatment are available. To measure whether guideline recommendations are implemented in daily practice, an instrument is needed that validly measures the quality of antibiotic use in daily clinical practice. Guideline-based quality indicators (QIs) are such tools. Recently, generic QIs for the antimicrobial treatment of bacterial infections in hospitals have been developed and validated [7]. The challenge is the embedding of appropriate antibiotic use, as defined by these QIs, in daily practice.

### Checklist

A checklist can be a tool for implementation; since it has been shown in other high-risk disciplines that adherence to checklists increases safety [8]. This also holds true for the field of medicine. For example, in surgery, implementation of a comprehensive multidisciplinary SURgical PAtient Safety System (SURPASS) is associated with an absolute risk reduction in surgical complications of 10.6% [9]. Similarly, the introduction of a checklist to improve patient care among gynaecologic oncology patients has resulted in a decreased length of stay of one day (4.5 days in the pre- and 3.5 days in the post-implementation period (P = 0.007)) [10]. In the field of infectious diseases, Pronovost et al. showed that introduction of a five-points checklist to reduce infections when inserting a central venous catheter, resulted in a decrease of the median rate of infections from 2.7 per 1,000 patients to zero after three months [11].

We hypothesize that an antibiotic checklist, to be used at the start of antibiotic treatment and after 48-72 hours of treatment, will improve clinical patient care, reduce costs and will ultimately contribute to the containment of antimicrobial resistance.

### Objectives

This study is set to implement an antibiotic checklist based on the validated generic quality indicators measuring the appropriateness of intravenously initiated antibiotic use in hospitalized adults treated for a suspected bacterial infection, and to analyse the effect of the introduction of the checklist on patient care in terms of shorter duration of intensive care unit (ICU)- and hospital stay, adequate treatment, decreased mortality rates, decreased total antibiotic use and lower costs.

## Methods and design

### Phase 1: The AB-checklist and barriers for use

The ‘Development of reliable generic quality indicators for the optimization of antibiotic use in the hospital’ (RIANT)-study used a RAND modified Delphi method to develop a set of generic QIs for appropriate antibiotic use in the treatment of all bacterial infections in hospitalized adult patients [7]. The clinimetric properties of these QIs have been tested in 1,890 hospitalized patients, in 22 Dutch hospitals. This analysis resulted in eight useful QIs, of which seven were process indicators that focus on the actual care provided. The antibiotic checklist includes these seven QIs.

**Figure 1:**
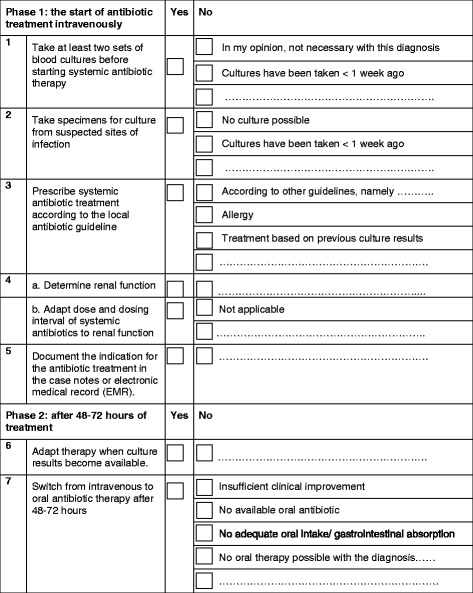
**The antibiotic checklist.**

The antibiotic checklist is intended as a reminding tool for the physician who starts intravenous (IV) antibiotic treatment. It is actually divided into two bundles (Figure 1). The first bundle has to be completed at the moment of prescribing IV antibiotics, and comprises five items. The second bundle is used during the course of treatment, at the latest after 72 hours of treatment. This part consists of two items.

As a preparation, we identified barriers of implementing this checklist, using a questionnaire based on an instrument of the Netherlands Organization for Applied Scientific Research (TNO), which is designed to survey determinants that influence the uptake of an innovation in health care organizations [12,13]. The questionnaire was sent to specialists and residents who work at the participating hospitals. Based on the identified barriers, the checklist design and its implementation format were adapted.

### Phase 2: cluster randomized clinical trial to evaluate the AB-checklist

#### Study design and setting

The (cost-) effectiveness of introducing the antibiotic checklist will be studied in a controlled, multicentre, prospective study using a stepped wedge design, comparing outcomes before and after implementation of the checklist while accounting for potential confounders [14].

**Figure 2:**
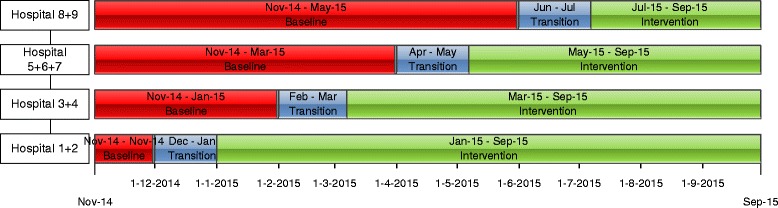
**The stepped wedge design for checklist implementation.**

The checklist will be introduced in nine hospitals over four time periods, during a total study period of eleven months (Figure 2). The aim is to include at least 810 patients in the period before- (the baseline group), and at least 810 patients with a completed checklist in the period after checklist introduction (intervention group). During the transition period implementation activities will be started and no patients will be included. This period runs from one month before introduction of the checklist to one week after introduction. Since compliance to the checklist will not be 100%, we will also collect data concerning the LOS from the patients in whom the checklist should have been but was not completed during the period after checklist introduction (the spill over group).

To promote selection of a patient population representative for every day clinical practice in the Netherlands, two university hospitals and seven teaching hospitals will participate, and in each hospital at least one surgical and one non-surgical ward will be included. The ICU and the paediatric ward will be excluded, as the quality indicators do not apply to these populations. Patients with a suspected bacterial infection presenting on the emergency department (ED) will be included if they are going to be hospitalized on one of the participating wards.

#### Intervention

The study coordinator will design a project plan together with the local project leader. This plan will focus on physicians, nurses and medical students who are involved in patient care. The plan will include standard items (see Table 1), but additional items can be added, if locally required, to cover practical issues as for example: How do we arrange that for each patient starting with antibiotics intravenously a checklist is filled in? How do we arrange that the checklist does not get lost?

**Table 1:** **Description of activities to stimulate use of the antibiotic checklist**

**Activity**	**Details**	**For whom**	**When or how**
**Education, including feedback**	About the need of appropriate antibiotic use and the room for improvement in current antibiotic use based on the baseline measurement; About the guidelines, adaption to renal function and IV-oral switch.	Physicians, medical students	Kick-off meeting; Website, e-learning
**Reminders**	Laminated pocket versions; posters	Physicians	During the whole intervention period
**Involvement of the whole health care team**	Involvement of a supervisor of each participating department in the implementation process	Physicians	Prior to implementation and during implementation
	Informing nurses about the study project	Nurses	e-mail, website, e-learning
	Informing medical students about the study project	Medical students	Letter, website, e-learning

### Patient enrolment

#### Eligible patients

**Figure 3:**
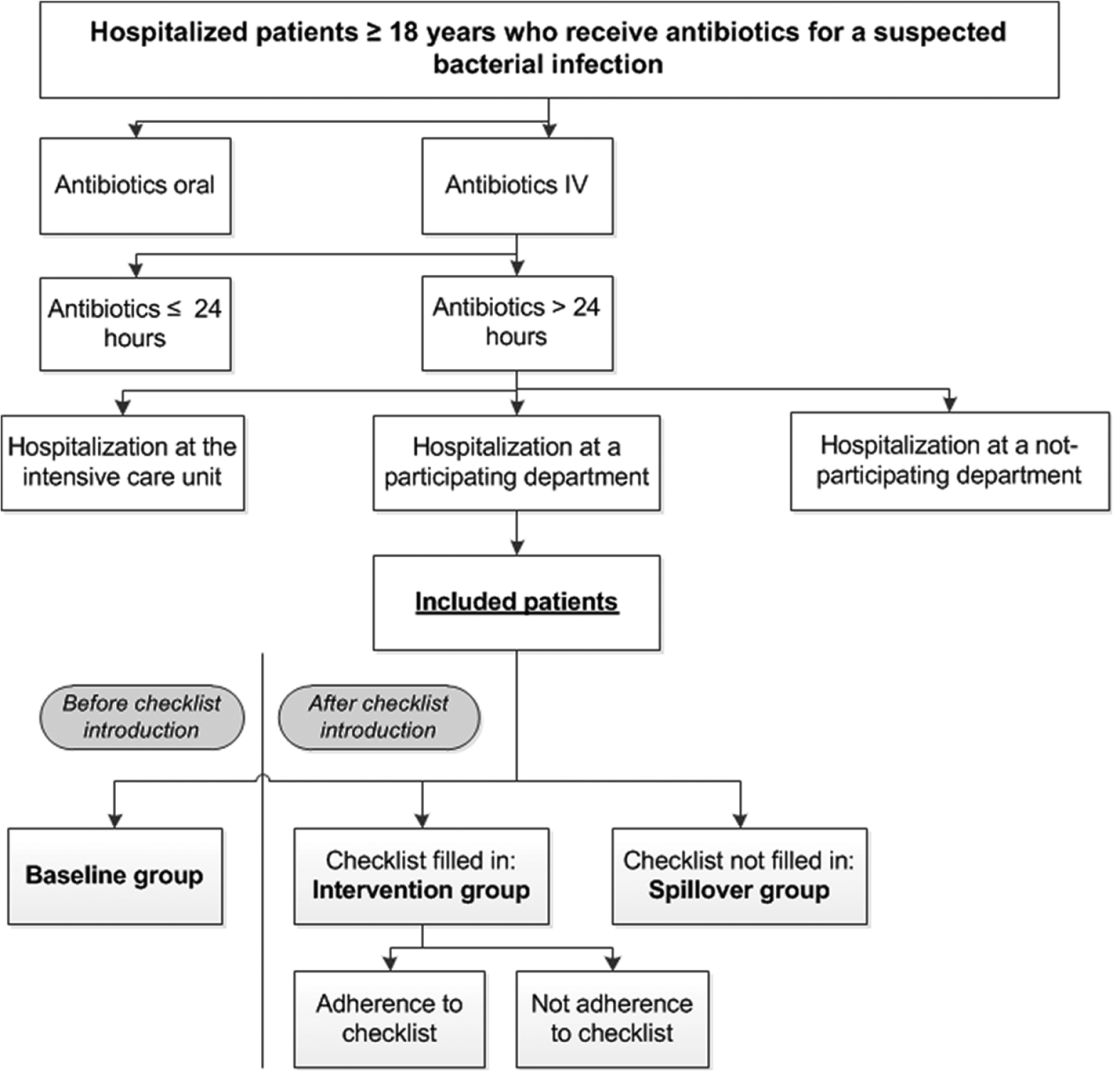
**Flowchart of the included patient in our study.**

The physician will use the checklist in all hospitalized adults (≥18 years old), or adults at the ED who will be admitted at a participating ward, with a suspected community-acquired and/or hospital-acquired bacterial infection, and will be treated with intravenous antibiotics. The treating physician identifies these patients upon prescription of antibiotic therapy. The patients included in the study are all patients that meet the above mentioned inclusion criteria, whether they have a completed checklist or not (see Figure 3 for the flowchart). They will be included in each hospital using an overview of the patients who started with intravenous antibiotics. This overview will be generated by the local departments of hospital pharmacy from the computerized physician order entry systems. Patients are excluded from the study in case of anticipated hospital stay of less than 24 hours, antibiotics used as prophylaxis or intended for less than 24 hours, hospitalization at the ICU at the start of the treatment, transfer from another hospital, or antibiotic treatment in the ambulance before presentation at the ED.

### Primary and secondary endpoints

The primary endpoint is length of hospital stay in days. LOS in community-acquired infections will be defined as the number of days between admission and discharge. In hospital-acquired infections LOS will be defined as the number of days between start of antibiotic treatment and discharge. Secondary endpoints are appropriate antibiotic treatment according to the generic QIs (yes/no per QI and sum score of all QIs) [6], admission to and duration of ICU stay in days, readmission within 30 days, mortality in the hospital and in the first 30 days after discharge, total antibiotic use, costs of hospital stay and costs associated with implementation.

### Assessments

For patient outcome we will collect data concerning the LOS, admission to and duration of ICU stay, readmission within 30 days and mortality in hospital or within 30 days after discharge for the baseline group and the intervention group. Furthermore we will collect data for possible confounders of patient’s outcome, namely age, sex, community-or hospital acquired infection, Charlson comorbidity index [15,16], use of antibiotics during the previous 30 days, and clinical condition as assessed by the Modified Early Warning System [17]. To calculate QI performance we will collect data concerning the number of taken blood cultures including its results, the number of taken cultures of suspected sides of infection including its results, antibiotic use including dose, interval and duration of treatment in days, the (suspected) type of infection and relevant laboratory parameters. For the spill over group, we will only collect data concerning the LOS and the possible confounders.

Antibiotics included in the study belong to group J01 (antimicrobials for systemic use) of the Anatomic Therapeutic Chemical (ATC) classification system. When multiple antibiotics are prescribed to one patient, all antibiotics will be registered. Antiviral therapy, medication for tuberculosis, antibiotics used for non-systemic selective intestinal decontamination and antibiotics used as pro-kinetics will be excluded.

### Sample size

The power analysis is based on previous results of appropriate antibiotic use on length of stay as described at ‘background’ [6,7]. In a simulation study based on the stepped wedge design, with nine hospitals each contributing 180 patients with a suspected infection, we estimated the power to approximate 80% to demonstrate a significant reduction of 13% in length of stay, assuming that the intraclass correlation coefficient does not exceed 0.20. With each hospital contributing 180 patients with a suspected infection, the total sample size will be 1620 patients. For each hospital we have determined a minimum number of patients in the baseline group and intervention group, depending on the randomization order of the hospital in the stepped wedge.

### Analysis

#### Effect evaluation

We will use mixed linear models to analyse the overall effect of the introduction of the checklist on length of stay. Therefore we will evaluate differences between baseline (before introduction of the checklist) and intervention plus spill over (after introduction of the checklist) (intention to treat analysis). We will also determine the theoretical effect of the checklist on LOS by evaluating differences between baseline and intervention data without the spill over data (per protocol analysis). Differences between the baseline data and the intervention data for secondary outcomes will also be evaluated with mixed linear models. For dichotomous outcomes (mortality), the comparison will be evaluated with a similar approach, using generalized estimating equation models. These mixed models account for within-cluster dependencies, and allow adjustment for possible confounders. To assess whether differences between these groups may be explained by confounding patient characteristics, we will first test with multivariate multilevel (linear mixed) analyses if these variables have a significant effect (p < 0.05) on the outcomes. Hereafter, we will compute the estimate of the total causal effect of checklist implementation on the outcomes before and after adjusting for a significant variable. If the estimate varies 10% or more, possible confounding is present and in that case we will adjust for this variable.

#### Validation and process evaluation

We will evaluate the physicians’ compliance with the checklist. Firstly we will check whether the ticks in the completed checklists are in accordance with the performance in clinical practice. Secondly we will determine the percentage of eligible patients for whom the checklist has been used after checklist introduction. The number of patients in whom the checklist was actually used (the intervention group) will be divided by the number of patients in whom it should have been used (the intervention plus the spill over group).

#### Economic evaluation

The key question for the economic evaluation is whether the benefits of using the checklist, which will likely include reduced antibiotic use, reduced length of stay and associated costs, justify the costs associated with implementation activities as well as daily use of the checklist. For reasons of feasibility, the cost analysis will only include a few main cost drivers of health care utilization, namely LOS on a general or ICU ward and antibiotic use. Unit costs for health care utilization will be estimated according to the Dutch guideline on (unit) costing in health care [18]. LOS and ICU stay will be valued based on the guideline prices. Furthermore, we will compare the total antibiotic use in days per antibiotic class in the baseline and the intervention group. Antibiotic costs will not be valued because the benefits become more evident in terms of reduced quantities rather than their particular costs. Implementation costs will be divided in non-recurring and recurring costs. Non-recurring costs are related to our study, such as costs of the development of the materials, the implementation strategy and costs of evaluation of the implementation. Recurring costs are the costs of accomplishment of the implementation strategy. The primary analysis is a cost-effectiveness analysis, with the incremental cost-effectiveness ratio expressed as the implementation related costs per reduction of length of stay in days. As we hypothesize that an effective implementation of the checklist will result in reduced health care use and thus lower costs, we will also perform a cost-benefit analysis, evaluating whether the implementation costs are offset by this cost reduction (costs of implementation < health care cost reduction). Robustness of the results for uncertainty in the assumptions will be evaluated in sensitivity analyses, including estimates of unit costs and volume of implementation activities (considering less activities will be needed if checklists become part of standard care). In a budget impact analysis results from the economic evaluation will be extrapolated to the national level to estimate the total clinical and budget impact in terms of health benefits, reduction in antibiotic use, and associated economic impact on the health care budget per annum for the Netherlands. The budget impact analysis will be performed according to the criteria provided by Mauskopf et al. [19], from a societal perspective as well as from the health insurance and/or national health care budgeting framework perspective. We will evaluate the impact of varying implementation rates, comparing care as usual (0% implementation), potential impact (100% implementation), and the probable rates of 85% and 50% implementation.

### Ethical considerations

The project proposal was discussed with the Medical Ethics Research Committee of the Academic Medical Centre. They confirmed that the Medical Research Involving Human Subjects Acts does not apply to this study and that an official approval by the committee was not required, because patients will receive treatment according to standard care, and will have no burden of checklist use.

## Discussion

This study protocol describes the design, implementation and evaluation of the AB-checklist study. To our knowledge, no other published studies or ongoing trials (http://apps.who.int/trialsearch/) investigate the implementation of a generic antibiotic checklist to be used for suspected bacterial infections in the hospital. If (cost) effective, the AB-checklist will provide physicians with a tool to support appropriate antibiotic use in adult hospitalized patients who start intravenous antimicrobials.

## Abbreviations

AB: Antibiotic
AMR: Antimicrobial resistance
ED: Emergency department
ICU: Intensive care unit
IV: Intravenous
LOS: Length of hospital stay
QI: Quality indicators
